# ADHD medicine consumption in Europe after COVID-19: catch-up or trend change?

**DOI:** 10.1186/s12888-024-05505-9

**Published:** 2024-02-09

**Authors:** Sophie Gimbach, Daniel Vogel, Roland Fried, Stephen V. Faraone, Tobias Banaschewski, Jan Buitelaar, Manfred Döpfner, Richard Ammer

**Affiliations:** 1 Data Science Hub, MEDICE Arzneimittel Pütter GmbH & Co. KG, 58638 Iserlohn, Germany; 2 Department of Statistics, TU Dortmund University, 44221 Dortmund, Germany; 3 Department of Psychiatry, SUNY Upstate Medical University, Syracuse, NY 13210 USA; 4 Medical Faculty Mannheim, Central Institute of Mental Health, Heidelberg University, 68159 Mannheim, Germany; 5 Department of Cognitive Neuroscience, Radboud University Medical Center, Nijmegen, 6500 HB The Netherlands; 6 Karakter Child and Adolescent Psychiatry, University Centre, Nijmegen, The Netherlands; 7 Department of Child and Adolescent Psychiatry and Psychotherapy, Medical Faculty, University of Cologne, 50969 Cologne, Germany; 8 Poliklinik und Innere Medizin, University Hospital Münster, 48149 Münster, Germany

**Keywords:** ADHD, COVID19, Pandemic, Pharmacoepidemiology, Time series forecasting

## Abstract

**Background:**

Although the COVID-19 pandemic and its implications have been associated with mental health services utilization and medication consumption, there is no longitudinal study on the long-term impact on ADHD medication use trends.

**Methods:**

This study examines the European ADHD medication consumption in 2020 to 2022 compared to the predicted consumption assuming the persistence of pre-pandemic trends. Predictions are calculated using Seasonal Autoregressive Integrated Moving Average (SARIMA) models.

**Results:**

While European ADHD medication sales recorded a drop in 2020, they returned to the predicted level in 2021, even slightly exceeding it. In 2022, we found a clear exceedance of the predicted level by 16.4% on average at country level. Furthermore, the increase in consumption growth in the post-pandemic period (2021–2022) compared to the pre-pandemic period (2014–2019) was significant in 26 of the 28 European countries under consideration.

**Conclusion:**

There is strong evidence of a trend change in the ADHD medicine consumption growth throughout Europe after the COVID-19 pandemic.

**Supplementary Information:**

The online version contains supplementary material available at 10.1186/s12888-024-05505-9.

## Background

A steady growth in the use of attention deficit hyperactivity disorder (ADHD) medications has been reported for the decades prior to the COVID-19 pandemic [5, 16, 23]. This was associated with an increase in the general awareness of ADHD among the public, practitioners, and psychotherapists alike. An important contributing factor was the broadening of the diagnostic criteria, e.g., the elimination of exclusion criteria such as autism spectrum disorder (ASD) in the DSM-5 released in 2013 [1]. Also, DSM-5 reduced the symptoms threshold for adults and the necessity of a childhood onset for an adult-ADHD diagnosis has become a matter of debate [4, 18]. The COVID-19 pandemic is associated with a break in this steady growth: We showed in a previous study that, globally, ADHD medicine consumption in 2020 was on average per country 6% lower than predicted based on pre-pandemic data [12]. However, several studies reported adverse effects of the pandemic on patients with ADHD in form of increased ADHD symptoms [6, 24, 25]. In fact, towards the end of 2021, the actual consumption slightly exceeded the predicted consumption without the occurrence of the pandemic [12]. Recent studies reported that the pandemic led to greater stress in children with ADHD in general [11], as well as medium-term impacts on children with ADHD of pandemic-related stress specifically [26]. Pre-pandemic studies found prolonged effects of stress exposure in form of worsened and persistent ADHD symptoms from childhood into young adulthood [14, 15]. Because no studies to date have investigated ADHD medication consumption in Europe after COVID-19, this study aimed to assess the long-term impact of the pandemic in the European ADHD medication market, particulary answering the question if the post-pandemic surge is only a catch-up effect or if the pandemic has indeed accelerated consumption growth in the long run. We examined changes in ADHD medicine consumption trends from pre-pandemic (2014 to 2019) to post-pandemic (2021 to 2022) in 28 European countries. We also measured the deviation of the actual consumption in 2022 from the prediction under non-pandemic conditions.

## Methods

### Data sources

We used quarterly data obtained from IQVIA Multinational Integrated Data Analysis System (IQVIA MIDAS®). IQVIA MIDAS data combine country-level data, healthcare expertise and therapeutic knowledge in 90 + countries to deliver data in globally standardized forms to facilitate multi-country analyses.

The database includes the sales of generic and brand products and does not contain individual-level data. Thus, institutional review board approval was not required. This study is based on sales volume data of ADHD medications from Quarter 1, 2014 to Quarter 4, 2022 of 28 European countries including information about the number of sold standard units, the strength, the active substances, and the sales value of each drug. Some of the substances included in the data set are also approved for other indications than ADHD treatment. However, in this study only sales data of medications specifically indicated for the treatment of ADHD (ATC class N06B) were evaluated.

Population estimates of each country were obtained from the UN World Population Prospects 2022 report [28]. Definitions of the defined daily dose (DDD), which is the assumed average maintenance dose per day for a drug used for its main indication in adults [29] were obtained by the World Health Organization (WHO) for each substance.

### Data analysis

As we do not observe the quantity of interest, consumption, directly, we use the sales data as proxy. It is plausible to assume that the error margin of medicine that is sold, but not consumed, remains fairly stable across regions and time, and does not distort the spatial and temporal analysis. We chose DDD per 1000 inhabitants per day as the main quantity of interest. Although individual doses vary, this number strongly correlates with the prevalence of diagnosed patients. Using the country-level sales volume data, the measure ‘DDD per 1000 inhabitants per day’ was calculated as following:

$$\mathrm{DDD}\:\mathrm{per}\;1000\;\mathrm{inhabitants}\;\mathrm{per}\;\mathrm{day}\:=\:\mathrm{sum}\left(1000\:\ast\:\left(\left(standard\mathit\;units\mathit\;\mathit\ast\mathit\;strength\right)/DDD\right)/population\right)/\#days\;$$

where *standard.units* are the number of sold standard units of each medication, *strength* is the dose of its active substance, *DDD* is the defined daily dose depending on the substance, *population* is the population estimate of the country at the time of sales and *#days* is the number of days in the time period. This measure is useful for comparison as it is standardized not only by time but also by population size.

We analyzed the European ADHD medicine consumption usage before and after the pandemic (2014 to 2022), per country and in total. Pre- and post-pandemic time trend coefficients were estimated by linear regression models using consumption data in DDD per 1000 inhabitants per day from 2014 to 2019 (pre-pandemic) and from 2021 to 2022 (post-pandemic) including an intercept, time of sales (linear trend) and quarters as regressors. The year 2020 was left out in the regression analyses to avoid distortions. We tested, for Europe in total and for each country, the one-sided hypothesis that the post-pandemic trend coefficient is not larger than the pre-pandemic trend coefficient. Rejection of this one-sided hypothesis provides evidence for a post-pandemic increase of the trend. Details on the test are given in the Supplement.

Additionally, we analyzed the differences in active substances of ADHD medication. They can be divided into stimulants (amfetamine, dexmethylphenidate, lisdexamfetamine, methylphenidate), which are recommended as first-line pharmacological treatment, and non-stimulants (atomoxetine and guanfacine), which are recommended for patients who cannot tolerate stimulants, do not benefit from them or who suffer from substance use disorders [9, 19]. The effects can last 3–5 h (short-acting) or 8–12 h (long-acting).

To further quantify the impact of the COVID-19 pandemic on ADHD medicine consumption, we forecasted the sales volume of the years 2020 to 2022 based on the national consumption trends until 2019. The impact was assessed by fitting a SARIMA model [10] and comparing its predictions to the actual sales volume. We used R [22], version 4.1.3, for data analysis.

## Results

### Increased growth of European ADHD medicine consumption after the pandemic

Before the pandemic, the annual ADHD medicine consumption growth in Europe was consistently around 5 to 6% (Table 1). However, from 2018 to 2019, we observed a higher increase of 9.7%, which decreased from 2019 to the pandemic affected year 2020 to 4.7%. In the following years, we observed remarkably higher growth rates of 12.7% in 2021 and 15.2% in 2022.

**Table 1:** Annual ADHD medicine consumption and percentage change in Europe, 2014-22. DDD = defined daily dose

	2014	2015	2016	2017	2018	2019	2020	2021	2022
**Consumption in DDD per 1000 inhabitants per day**	1.85	1.95	2.07	2.17	2.30	2.52	2.64	2.98	3.43
**Relative change**		5.43%	6.18%	4.85%	5.97%	9.73%	4.74%	12.66%	15.17%

**Fig. 1:**
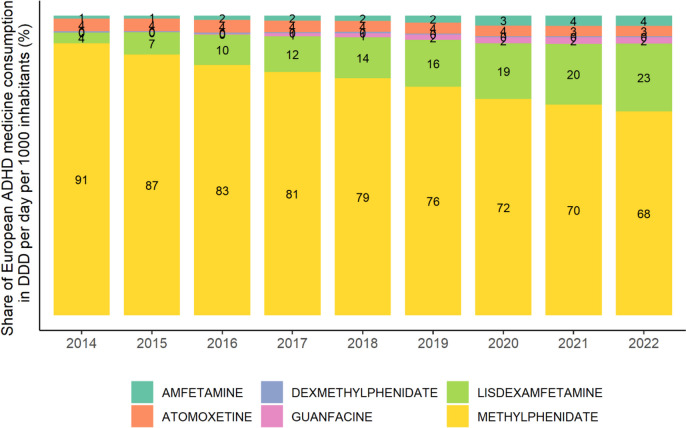
Share of substances on European ADHD medication consumption. DDD = defined daily dose

Comparing ADHD medication consumption by active substances (Table 2), we found increasing trends in all substances. Stimulants (methylphenidate, amfetamine, lisdexamfetamine or dexamfetamine) are by far the most frequently prescribed medications, followed by non-stimulants (atomoxetine or guanfacine). Methylphenidate has the highest share of the European consumption, however, due to rapid growth of lisdexamfetamine medications, its share decreases steadily (Fig. 1). We also found that consumption of long-acting medications increased stronger than prescriptions of short-acting medications, which accounted for 21% of total European consumption in 2014, but for only 15% in 2022 (Table 3).

**Table 2:** Annual ADHD medicine consumption by substance in Europe, in DDD per 1000 inhabitants per day. DDD = defined daily dose

Substance	2014	2015	2016	2017	2018	2019	2020	2021	2022
AMFETAMINE	0.020	0.022	0.032	0.037	0.042	0.060	0.089	0.105	0.120
ATOMOXETINE	0.077	0.081	0.087	0.086	0.086	0.094	0.094	0.103	0.117
DEXMETHYLPHENIDATE	0.006	0.007	0.007	0.008	0.008	0.008	0.009	0.010	0.011
GUANFACINE	0.000	0.000	0.005	0.020	0.033	0.044	0.054	0.064	0.074
LISDEXAMFETAMINE	0.066	0.142	0.209	0.259	0.312	0.392	0.488	0.602	0.773
METHYLPHENIDATE	1.657	1.674	1.710	1.755	1.812	1.918	1.901	2.085	2.325

**Table 3:** Annual ADHD medicine consumption by duration of action in Europe, in DDD per 1000 inhabitants per day. DDD = defined daily dose

	2014	2015	2016	2017	2018	2019	2020	2021	2022
Long-Acting	1,441	1,544	1,657	1,771	1,896	2,094	2,190	2,490	2,900
Short-Acting	0,386	0,382	0,393	0,393	0,397	0,423	0,446	0,479	0,519

**Fig. 2:**
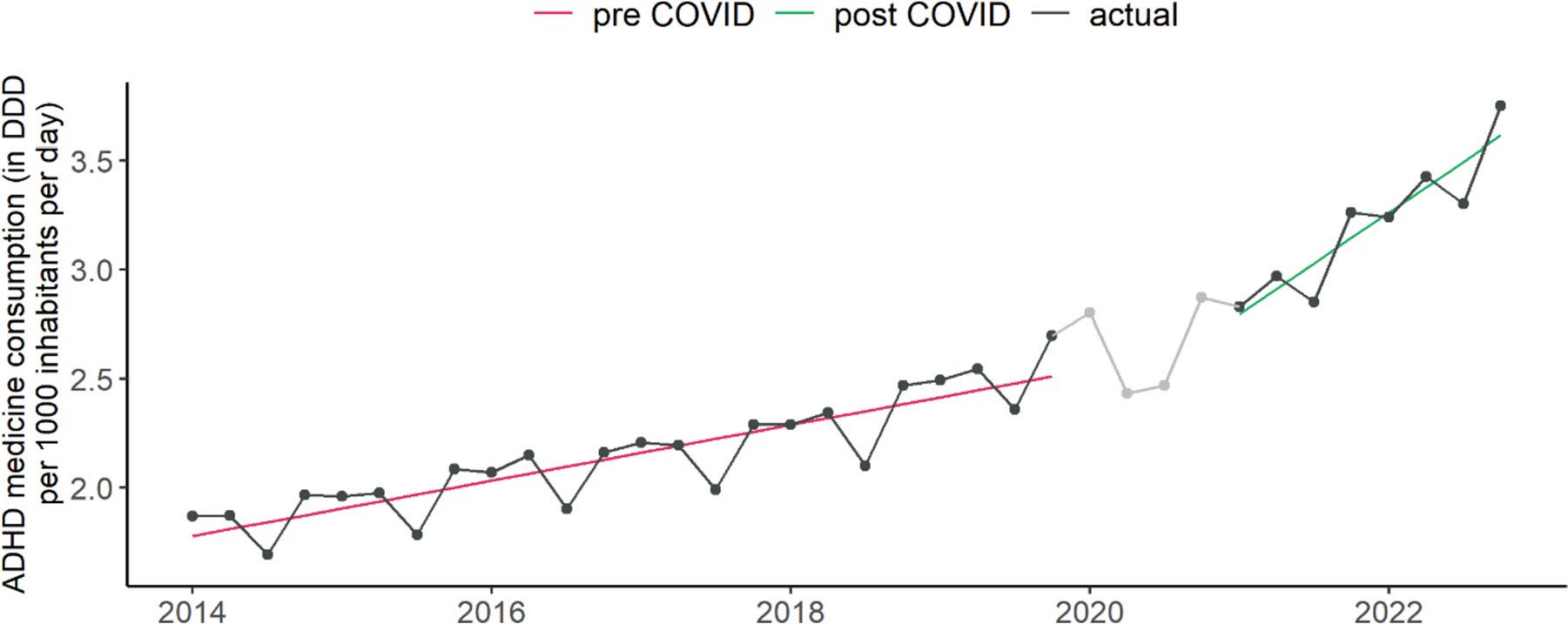
Quarterly ADHD consumption in Europe. Regression line in pre COVID years (2014–2019) is marked as red line, in post COVID years (2021–2022) as green line. DDD = defined daily dose

In line with previous studies, we found substantial differences in national ADHD medication use levels in 2019, ranging from 0.009 DDD per 1000 inhabitants per day in Bulgaria to 16.49 in Sweden. In general, the highest levels of medicine consumption were recorded in Scandinavian countries (Sweden, Norway, and Denmark), followed by the Netherlands, Switzerland, and Finland (Figure S2 in the Supplement). Eastern European countries generally showed the lowest consumption levels. However, time trends were comparable across most European countries. In the pre-pandemic years up to 2019, time trend estimates were significant at the 5% level in all countries except Romania and Luxembourg. In the post-pandemic period (2021 to 2022), regression slope estimates were significantly positive in all 28 countries. Moreover, post-pandemic time trend estimates were larger than pre-pandemic in all countries (Table 4; see also Figure S3 in the Supplement). In Luxembourg, and Romania, the consumption trend even switched from declining into increasing during the pandemic. In 26 of the 28 countries, the regression slopes after the pandemic (2021 to 2022) were significantly larger than before the pandemic (2014 to 2019). Non-significant increases were observed in Belgium and Croatia (Table 4). Throughout Europe (Fig. 2), the pre-pandemic regression slope was estimated as 0.03 (*p* < 0.001), increasing to 0.11 (*p* < 0.001) in the post-pandemic period. The latter is significantly larger than the former (*p* < 0.001). The individual-country analogues of Fig. 2 can be found the Supplement (Figure S2).

**Table 4:** Estimated parameters by linear regressions, and t-test *p*-values testing post-pandemic < = pre-pandemic

Country	Regression slope pre-pandemic	Regression slope post-pandemic	*p*-value one-sided t-test
Austria	0.014	0.068	0.000
Belgium	0.047	0.073	0.068
Bulgaria	0.000	0.001	0.013
Croatia	0.001	0.002	0.111
Czech Republic	0.015	0.030	0.000
Denmark	0.197	0.667	0.000
Estonia	0.022	0.182	0.000
Finland	0.159	0.609	0.000
France	0.015	0.050	0.000
Germany	0.033	0.110	0.000
Greece	0.003	0.012	0.000
Hungary	0.006	0.016	0.000
Ireland	0.030	0.102	0.000
Italy	0.003	0.004	0.000
Latvia	0.004	0.023	0.000
Lithuania	0.003	0.016	0.000
Luxembourg	-0.020	0.103	0.000
Netherlands	0.042	0.270	0.000
Norway	0.236	0.694	0.000
Poland	0.008	0.059	0.000
Portugal	0.015	0.164	0.000
Romania	-0.001	0.011	0.002
Slovakia	0.003	0.009	0.000
Slovenia	0.008	0.037	0.000
Spain	0.021	0.069	0.000
Sweden	0.292	0.588	0.000
Switzerland	0.060	0.243	0.000
UK	0.043	0.179	0.000

### Consumption of ADHD medicines exceeds predictions after COVID

**Fig. 3:**
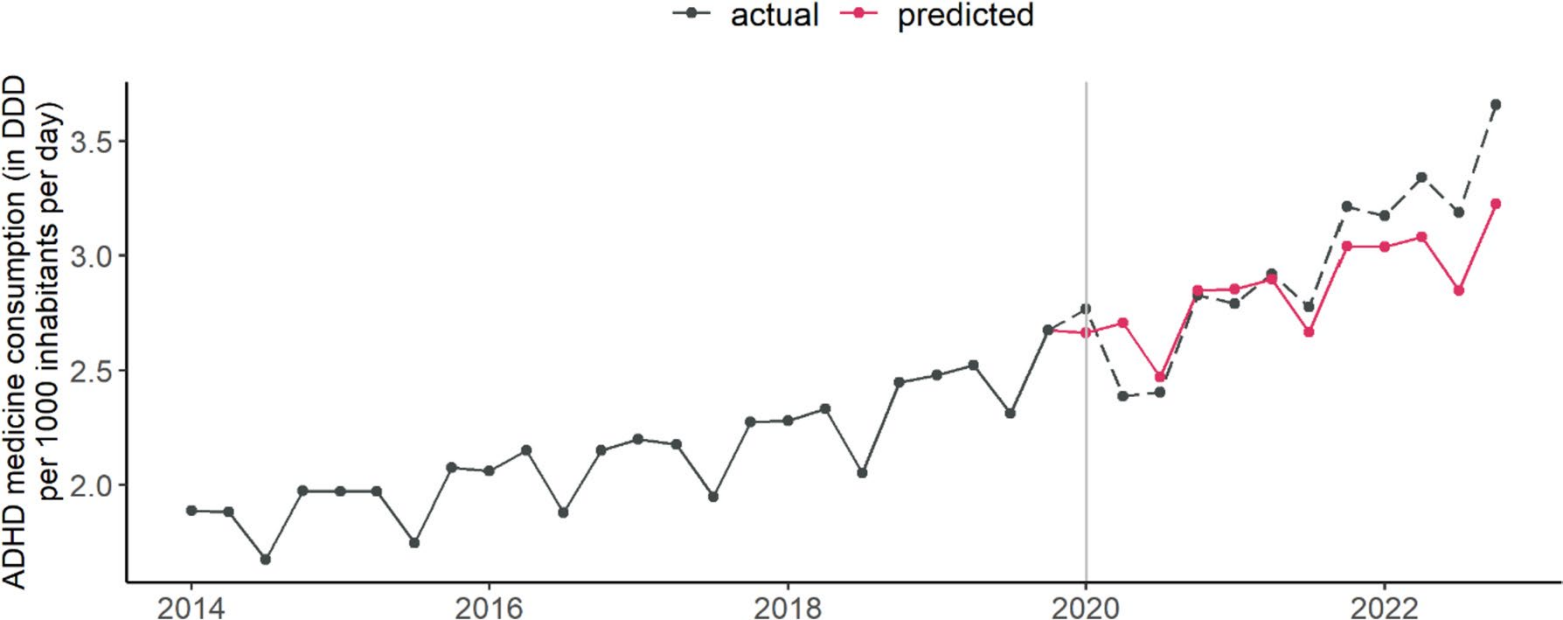
Predicted and actual global quarterly consumption of ADHD medication. DDD = defined daily dose

For each country and for Europe in total, we calculated the difference between the actual ADHD medication consumption for the period from 2020 to 2022 and the predicted consumption under non-pandemic conditions. Towards this end, seasonal autoregressive integrated moving average (SARIMA) models of order [1])× [1])_4_ were fitted using the maximum-likelihood method to the quarterly consumption during 2014 Q1 to 2019 Q4. Based on the thus fitted models, quarterly forecasts for the consumption levels in 2020, 2021, and 2022 were obtained. Hence, the pre-pandemic trends in national ADHD medicine consumption were extrapolated under the assumption of unchanged conditions, i.e., without the occurrence of the COVID-19 pandemic, see Fig. 3.

**Fig. 4:**
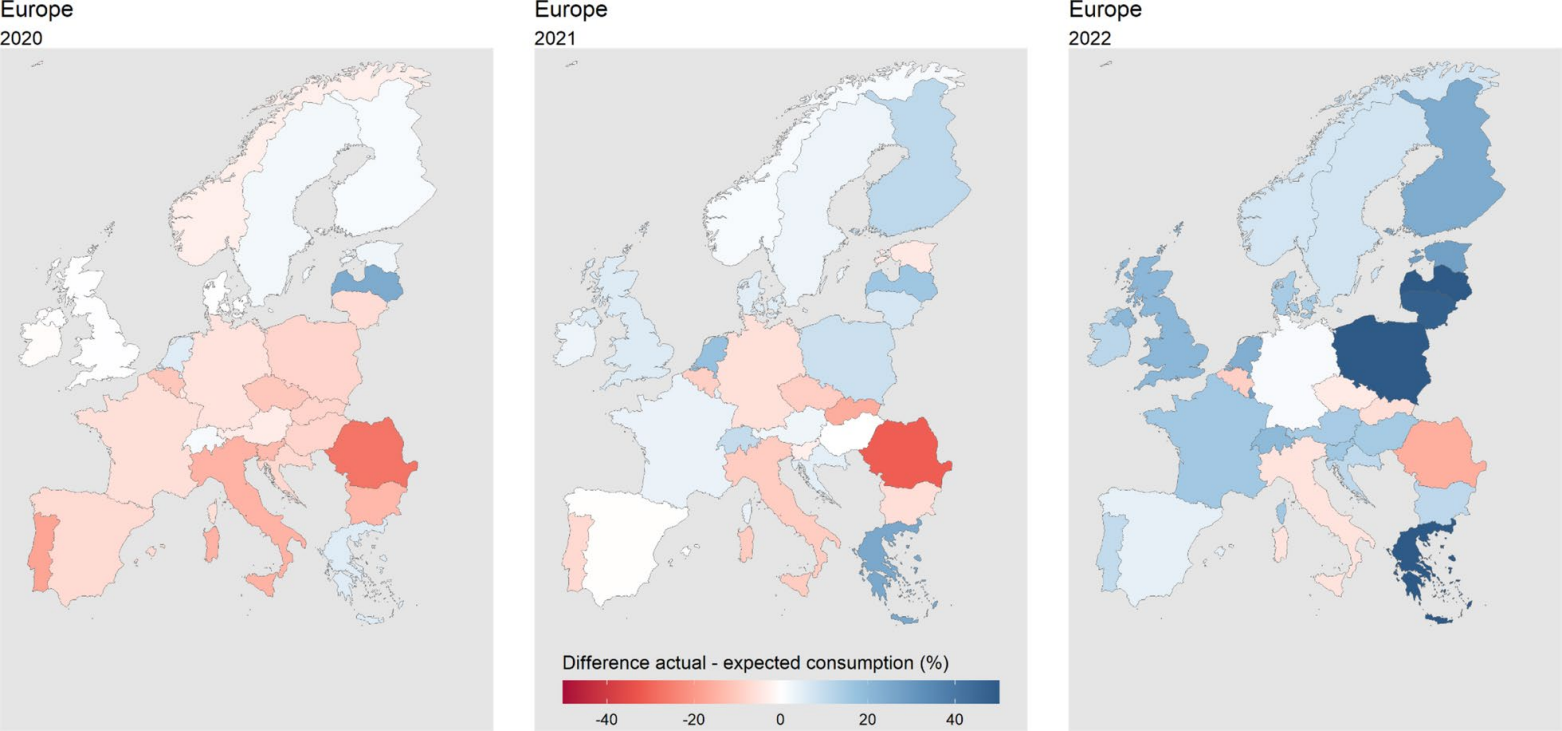
Percentage difference of actual and predicted ADHD medication consumption in 28 European countries in DDD per 1000 inhabitants per day in 2020, 2021 and 2022. DDD = defined daily dose

In the first year of the pandemic, we found lower consumption than predicted in most of the European countries. Twenty of the 28 European countries recorded a drop of ADHD medication consumption in 2020 (Table 5; Fig. 4). The highest losses were in Romania, Portugal, and Italy. Only in three countries (Latvia, Greece, and the Netherlands) did consumption substantially exceed the predictions. In Europe, ADHD medicine consumption amounted to 2.64 instead of the predicted 2.71 DDD per 1000 inhabitants per day, which corresponds to a relative loss of 2.34%. On country average, the national ADHD consumption in 2020 was 4.8% lower than predicted.

**Table 5:** Actual and predicted annual ADHD medicine consumption and relative difference per country in Europe

	2020			2021			2022		
Country	Actual consumption	Predicted consumption	% difference	Actual consumption	Predicted consumption	% difference	Actual consumption	Predicted consumption	% difference
AUSTRIA	1.07	1.11	-3.57%	1.26	1.22	2.64%	1.53	1.33	14.74%
BELGIUM	3.58	4.03	-11.02%	3.92	4.33	-9.32%	4.22	4.62	-8.77%
BULGARIA	0.01	0.01	-13.9%	0.01	0.01	-5.94%	0.01	0.01	9.43%
CROATIA	0.03	0.04	-7.44%	0.05	0.04	4.6%	0.05	0.05	9.66%
CZECH REPUBLIC	0.80	0.90	-10.65%	0.88	0.97	-9.42%	1.00	1.04	-4.12%
DENMARK	13.36	13.38	-0.17%	15.36	14.63	5.02%	18.03	15.69	14.95%
ESTONIA	1.00	0.97	2.49%	1.24	1.30	-4.21%	1.97	1.55	27.18%
FINLAND	6.71	6.62	1.37%	8.62	7.76	10.99%	11.05	8.90	24.16%
FRANCE	0.86	0.91	-6.27%	1.02	0.99	2.79%	1.22	1.07	14.12%
GERMANY	3.15	3.33	-5.49%	3.33	3.54	-5.85%	3.77	3.73	1.29%
GREECE	0.12	0.12	5.22%	0.16	0.13	25.18%	0.21	0.14	50.45%
HUNGARY	0.20	0.22	-8.15%	0.25	0.25	0.16%	0.31	0.27	14.52%
IRELAND	1.81	1.82	-0.53%	2.08	2.01	3.22%	2.49	2.22	12.36%
ITALY	0.07	0.09	-14.8%	0.09	0.10	-9.83%	0.11	0.12	-5.68%
LATVIA	0.15	0.12	24.33%	0.16	0.14	16.1%	0.26	0.16	62.3%
LITHUANIA	0.09	0.10	-6.54%	0.12	0.11	7.05%	0.19	0.13	46.16%
LUXEMBOURG	2.15	2.24	-4.17%	2.27	2.17	4.56%	2.68	2.09	28.02%
NETHERLANDS	9.56	9.09	5.2%	11.16	9.49	17.54%	12.24	9.90	23.66%
NORWAY	14.49	14.94	-3.01%	17.10	16.84	1.54%	19.88	18.45	7.76%
POLAND	0.31	0.34	-8.17%	0.40	0.37	8.87%	0.64	0.40	59.43%
PORTUGAL	2.27	2.75	-17.45%	2.71	2.91	-6.91%	3.36	3.05	10.31%
ROMANIA	0.13	0.18	-27.16%	0.13	0.20	-31.27%	0.18	0.21	-16.08%
SLOVAKIA	0.18	0.20	-8.34%	0.18	0.21	-15.9%	0.22	0.23	-6.25%
SLOVENIA	0.52	0.60	-13.22%	0.61	0.63	-2.94%	0.76	0.66	15.78%
SPAIN	3.18	3.41	-6.74%	3.51	3.50	0.14%	3.78	3.59	5.21%
SWEDEN	18.05	17.67	2.12%	19.47	18.87	3.15%	21.82	20.07	8.71%
SWITZERLAND	6.20	6.12	1.37%	7.01	6.38	9.9%	7.98	6.63	20.4%
UK	2.96	2.95	0.24%	3.32	3.15	5.57%	4.04	3.34	20.86%
Europe	2.64	2.71	-2.34%	2.98	2.90	2.65%	3.43	3.08	11.52%

In 2021, ADHD medication consumption in European countries on average returned back to the predicted level (Table 5; Fig. 4). While ten countries still recorded lower actual sales than forecasted, the remaining 18 countries met or even exceeded predictions. On average, the actual national ADHD medicine sales in 2021 were 0.98% above their predictions. Highest losses were recorded in Romania, Slovakia, and Italy, whereas Latvia, Greece, and the Netherlands again surpassed predictions the most. Throughout Europe, the actual consumption in 2021 (2.98 DDD per 1000 inhabitants per day) was 2.65% higher than predicted (2.90 DDD per 1000 inhabitants per day).

In 2022 however, medication usage levels were not only close to pre-pandemic predictions, but clearly exceeded them (Table 5; Fig. 4). In 23 of the 28 countries, the actual ADHD medication consumption was higher than predicted; in ten countries consumption surpassed predictions by more than 20%. The average relative deviation revealed a mean exceedance of national consumption forecasts by 16.4% in 2022. Only in five countries ADHD medicine sales were below the predictions. Belgium (-9%), Slovakia (-6%), the Czech Republic (-4%), Romania (-16%), and Italy (-6%). These countries were also among those most negatively impacted in 2020 and 2021.

Throughout Europe, the consumption in 2022 was predicted as 3.08 DDD per 1000 inhabitants per day without the emergence of COVID-19, whereas the actual consumption amounted to 3.43 DDD per 1000 inhabitants per day, which corresponds to an increase of 11.5%.

**Fig. 5:**
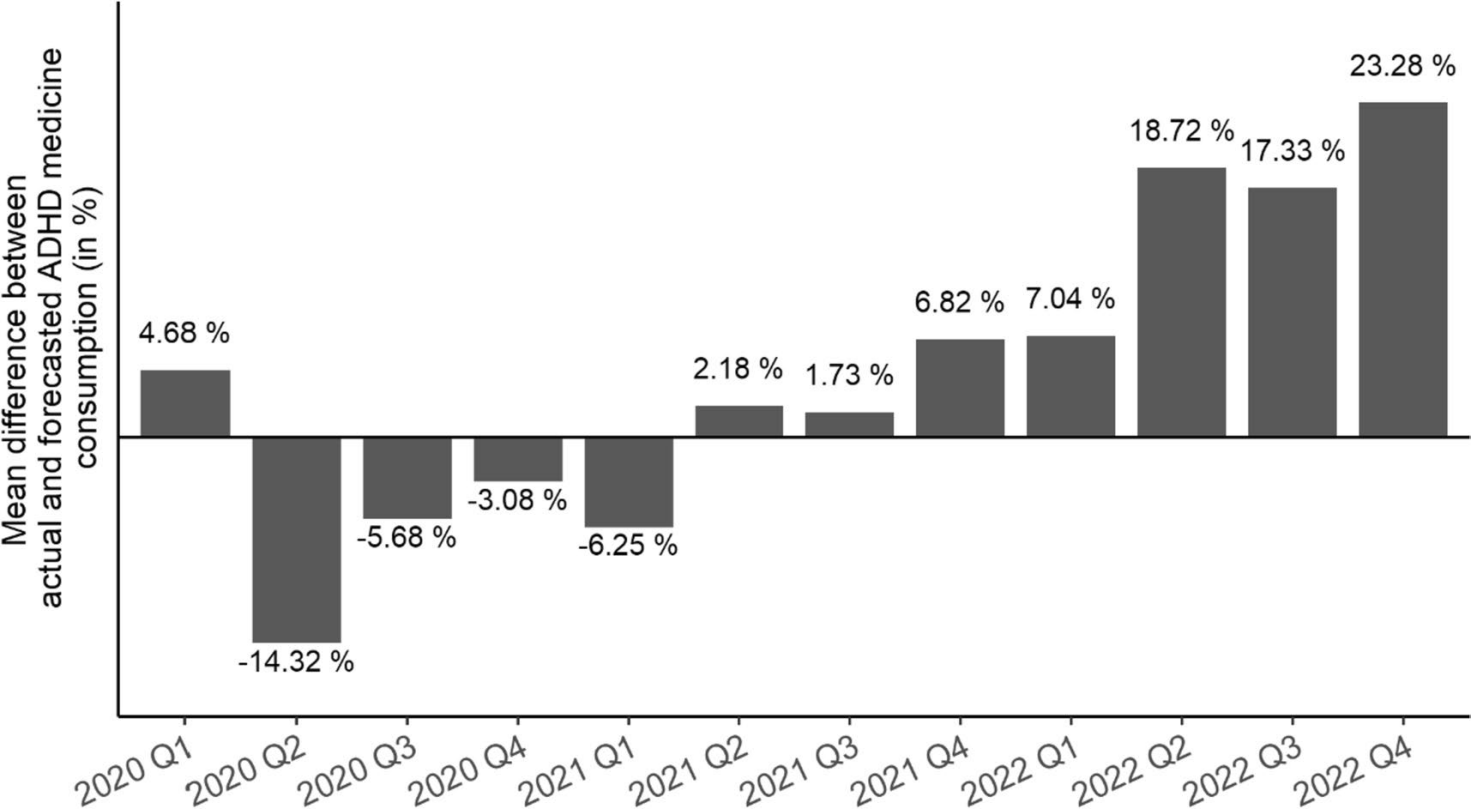
Mean relative difference between actual and predicted ADHD medicine consumption (in DDD per 1000 inhabitants per day) per quarter, 2020–2022. DDD = defined daily dose

Comparing the mean national (unweighted averages) relative differences per quarter (Fig. 5), we found remarkably high exceedances of the predictions in the last three quarters of 2022. While in the first quarter of 2022, the actual consumption levels were on average 7% above forecast, which is about the same extent as the last quarter in 2021, consumption in the second and the third quarters surpasses the pre-pandemic trends by 18.7% and 17.3%, respectively. In Quarter 4 of 2022, the actual consumptions were on average even 23.3% higher than predicted, which contradicts the assumption of the increased ADHD medication use being only a catch-up effect of 2020.

## Discussion

In this study, we found an increased consumption growth of ADHD medications in Europe after the pandemic. A possible cause is the general increase of mental health issues caused by anti-pandemic mitigation measures, including the worsening of ADHD symptoms [6, 21]. Recent research showed a medium-term impact of pandemic-related stress on children with ADHD [26]. Social withdrawal is also associated with a decreased likelihood of symptom remission in patients with major psychiatric disorders [20], which potentially led to greater ADHD medication demand in the long run. However, other factors unrelated to the pandemic might have also been contributing to the rise in ADHD medication sales. Changing diagnosis criteria as well as increasing awareness of ADHD in media and social media, indicated, e.g., by a steadily increasing popularity of the Google search term “ADHD” [13], led to a significantly increased prevalence in recent years, especially in individuals of colour and females [1]. Although the role of social media and “ADHD influencer” are a relatively new phenomenon, the number of views on TikTok of the hashtag #ADHD was frequently reported as 11 billion as of May 2022 [17] and has reached 33 billion now [27]. Also, access to an ADHD diagnosis has become easier. In several countries, the initial referral to a diagnosis can now be carried out be a GP rather than a psychotherapist [2, 3]. This is a response to the high demand of psychiatric support in comparison to the limited resource, resulting in long waiting times to diagnoses and therapies. Both of these effects, social media and easier access, potentially have been amplified by the pandemic, where access to healthcare resources was limited, but the psychological burden of social isolation and the time spent on social media largely increased.

In line with our results, other studies analyzing the impact of the pandemic on medication use found a significant increase in the consumption of psychotropic drugs in the French population after March 2020 [7] and an increase in rates of psychotropic medication use and psychiatric disorder diagnoses for Danish youths from March 2020 to June 22 [8]. We have also carried out analogous analysis for 17 other countries in different regions of the world. With four notable exceptions (e.g. China, Indonesia), a very similar picture emerges throughout the world. However, as we do not have complete data coverage of the world, most notably, not of North America, which is by far the biggest market of ADHD medicine, this study focused on Europe. Although Europe comprises different cultural areas with quite diverse histories and perceptions of ADHD and strong differences in the diagnosis prevalence, the increase in the consumption trend was observed throughout all considered European countries. This provides evidence that this is not a localized effect that can be attributed to cultural or healthcare-system-specific reasons.

In this study, the use of international pharmaceutical sales data enabled an analysis and quantification of the medium- and long-term effect of the COVID-19 pandemic response measures on ADHD medication consumption in Europe. However, the prevalence of ADHD was not examined as this study only evaluates sales data of ADHD medications. Furthermore, the data do not contain individual-level treatment data necessary to measure trends by age, gender, or appropriateness of prescribing. Therefore, we cannot draw any conclusions regarding overuse, underuse, or misuse.

## Conclusion

This study examined the medium-term impact of the COVID-19 pandemic on ADHD medication use by analyzing sales data of 28 European countries from 2014 to 2022. Consistent with previous research [5, 23], we observed a wide variation in ADHD medication use, with the highest usage levels in Northern countries and lower levels in Eastern Europe. However, all countries except Luxembourg and Romania recorded an increasing trend in linear regression models significant at the 5% level in the pre-pandemic period from 2014 to 2019. After a drop in 2020, especially in the second quarter, when social mitigation measures were taken to prevent the spread of the at the time novel Coronavirus, medication consumption returned to the predicted level by 2021 in most countries. Towards the end of 2021, usage levels even tended to exceed predictions. Throughout Europe, the actual consumption was 2.3% below predictions in 2020. In contrast, in 2021, consumption surpassed pre-pandemic predictions by 2.6%. While these exceedances in 2021 might be a catch-up effect due to reduced consumption in previous quarters, the noticeably increased growth observed in all four quarters of 2022 argues in favour of the pandemic having a catalyzing effect on ADHD medication consumption growth in the long run. Throughout Europe, the annual ADHD medicine consumption, predicted as 3.08 DDD per 1000 inhabitants per day, achieved an actual value of 3.43 DDD per 1000 inhabitants per day, corresponding to a relative rise of 11.5%. At country-level, 23 of the 28 European countries recorded higher ADHD medication sales volumes than predicted, ten of them even surpassed expectations by more than 20%. On average, national annual consumption was 16.4% higher than predicted. Quarterly comparisons of predictions and actual values revealed relatively small deviations in Quarter 1 of 2022 but increasing positive deviations in the following quarters with Quarter 4 showing the highest mean national exceedance of forecasts by more than 23%.

In all European countries examined, the estimated regression time trend parameter in the post-pandemic period (2021 to 2022) was higher than in the pre-pandemic period (2014 to 2019). Decreasing trends in Romania and Luxembourg were reversed after the pandemic. In 26 of the 28 countries, the post-pandemic time trend was significantly larger than before the pandemic. In the European total, the slope estimator rose significantly (*p* < 0.001) from 0.03 in the pre-pandemic period to 0.11 in the post-pandemic period.

Further research is needed to investigate the long-term impact of the pandemic on ADHD prevalence and prescribing patterns as well as medication trends beyond the study period. Moreover, analyses of this study should also be carried out for countries in other continents, to examine if this is a European trend only or if similar effects can be observed globally. Health care systems should build strategies that can prevent population at risk from long term effects caused by socially disruptive events like the pandemic effectively.

### Supplementary Information

**Additional file 1.**

## Data Availability

The data that support the findings of this study are available from IQVIA but restrictions apply to the availability of these data, which were used under license for the current study, and so are not publicly available. Data are however available from the authors upon reasonable request and with permission of IQVIA. Please note that if you do wish to share your raw data and do not have consent from all patients to publish this data, it will need to be de-identified.

## Abbreviations

ADHD: Attention Deficit Hyperactivity Disorder
DDD: Defined Daily Dose
SARIMA: Seasonal Autoregressive Integrated Moving Average
WHO: World Health Organization
