# Supplementary material for: ADHD medicine consumption in Europe after COVID-19: catch-up or trend change?
Source: BMC Psychiatry. 2024 Feb 9;24:112. doi: 10.1186/s12888-024-05505-9 (PMC10854136; doi:10.1186/s12888-024-05505-9)
Supplement: Supplementary file 1 — Additional file 1. [file 12888_2024_5505_MOESM1_ESM.pdf]

Supplementary Material to

ADHD medicine consumption in Europe after COVID-19: catch-up or trend change?

by Sophie Gimbach, Daniel Vogel, Roland Fried, Stephen V. Faraone, Tobias Banaschewski, Jan Buitelaar, Manfred Döpfner, and Richard Ammer

Contents

1 Technical Appendix: Testing the equality of two regression slopes when a seasonal pattern is present 2
1.1 Testing simple linear hypotheses within linear regression models 2
1.2 Testing the equality of regression slopes – ignoring the seasonal pattern 2
1.3 Testing the equality of regression slopes – including the seasonal pattern 3

2 Further Figures 5
2.1 Consumption levels in 28 European countries in 2019 5
2.2 Consumption growth in 28 European countries from 2014 to 2022 6

3 Data source 8

1 Technical Appendix: Testing the equality of two regression slopes when a seasonal pattern is present

1.1 Testing simple linear hypotheses within linear regression models

We consider the following regression model:

$$Y = X\beta + \epsilon,$$

i.e.,

$$\begin{pmatrix} y_1 \\ \vdots \\ y_n \end{pmatrix} = \begin{pmatrix} x_{11} & \dots & x_{1p} \\ \vdots & & \\ x_{n1} & \dots & x_{np} \end{pmatrix} \begin{pmatrix} \beta_1 \\ \vdots \\ \beta_p \end{pmatrix} + \begin{pmatrix} \epsilon_1 \\ \vdots \\ \epsilon_n \end{pmatrix},$$

where the y_i are the observations, $x_{i,j}$ are non-random and known (the design matrix), the β_j are the unknown (non-random) parameters, and $\epsilon_i \sim N(0, \sigma^2)$ are independent. We test the simple (i.e. one equation) hypothesis $H_0 : r^\top \beta = r_0$ for some $r \in \mathbb{R}^p$, $r_0 \in \mathbb{R}$ by means of the following t test statistic:

$$\frac{r^\top \hat{\beta} - r_0}{\sqrt{\hat{\sigma}^2 r^\top (X^\top X)^{-1} r}} \overset{H_0}{\sim} t_{n-p}, \tag{1}$$

where $\hat{\beta} = (X^\top X)^{-1} X^\top Y$, $\hat{\sigma}^2 = \frac{1}{n-p} \sum_{i=1}^n \hat{\epsilon}_i$, and $\hat{\epsilon} = Y - X\hat{\beta}$ is the vector of residuals [e.g. Lehmann and Romano, 2005, Chapter 7]. In case of more than one equation, the general formulation of the linear-hypothesis test is by means of an F test statistic, an example being ANOVA. One advantage of the t test formulation in the univariate case is that we can perform one-sided tests. This is indeed of interest here. We deliberately test the one-sided hypothesis that the slope after the pandemic is *not larger* than before, the rejection of which provides evidence for an actual increase of the consumption growth. If the slope after the pandemic was lower than before, this would conversely support the impression of a catch-up effect.

Interestingly, the two-sample case is already contained in the above formulation. If we have two independent regression models

$$Y^{(1)} = X^{(1)} \beta^{(1)} + \epsilon^{(1)} \qquad \text{and} \qquad Y^{(2)} = X^{(2)} \beta^{(2)} + \epsilon^{(2)}$$

with the same parameter dimension p, the same error variance σ^2, but potentially different sample sizes n_1 and n_2, respectively, they can be put together in one model

$$Y = X\beta + \epsilon,$$

with $Y = (Y^{(1)}, Y^{(2)})$, $\beta = (\beta^{(1)}, \beta^{(2)}) \in \mathbb{R}^p$, $\epsilon = (\epsilon^{(1)}, \epsilon^{(2)})$, and design matrix

$$X = \begin{pmatrix} X^{(1)} & 0 \\ 0 & X^{(2)} \end{pmatrix} \in \mathbb{R}^{(n_1+n_2) \times 2p}.$$

Strictly speaking, we do not have two samples here, but rather want to different periods of the time series. But since we are assuming independent errors, this is the same situation. Also, this setting makes the same-error-variance assumption even more plausible.

1.2 Testing the equality of regression slopes – ignoring the seasonal pattern

Consider the data of Figure 1 in the main document, i.e., the quarterly DDD per day and 1000 inhabitants in 28 European countries, from 2014 to 2022, but excluding the 2020, i.e., we have the vector of observations $Y = (Y^{(1)}, Y^{(2)})$ where $Y^{(1)} = (y_1, \dots, y_{24})$ corresponds to the 6 years 2014 to 2019, and $Y^{(2)} = (y_{25}, \dots, y_{32})$ to the 2 years 2021 to 2022. We fit the regression model

$$\begin{pmatrix} Y^{(1)} \\ Y^{(2)} \end{pmatrix} = \begin{pmatrix} y_1 \\ y_2 \\ \vdots \\ y_{24} \\ y_{25} \\ \vdots \\ y_{32} \end{pmatrix} = \begin{pmatrix} 1 & 1 & 0 & 0 \\ 1 & 2 & 0 & 0 \\ \vdots & \vdots & \vdots & \vdots \\ 1 & 24 & 0 & 0 \\ 0 & 0 & 1 & 1 \\ \vdots & \vdots & \vdots & \vdots \\ 0 & 0 & 1 & 8 \end{pmatrix} \begin{pmatrix} \beta_{ic}^{(1)} \\ \beta_{sl}^{(1)} \\ \beta_{ic}^{(2)} \\ \beta_{sl}^{(2)} \end{pmatrix} + \begin{pmatrix} \epsilon_1 \\ \epsilon_2 \\ \vdots \\ \epsilon_{24} \\ \epsilon_{25} \\ \vdots \\ \epsilon_{32} \end{pmatrix}, \tag{2}$$

where $\beta_{ic}^{(1)}$, $\beta_{sl}^{(1)}$ are intercept and slope parameter, respectively, before the pandemic, and $\beta_{ic}^{(2)}$, $\beta_{sl}^{(2)}$ after the pandemic. The starting point of the count in the fourth column of the design matrix is arbitrary. It may as well continue at 25. This only affects interpretation of $\beta_{ic}^{(2)}$, which is not of interest here. The hypothesis of interest is H_0: $\beta_{sl}^{(2)} \leq \beta_{sl}^{(1)}$, i.e., $r = (0, -1, 0, 1)$ and $r_0 = 0$ in the phrasing of (1). We obtain

$\hat{\beta}_{sl}^{(1)}$	$\hat{\beta}_{sl}^{(2)}$	$\hat{\sigma}^2$	t-value (test statistic)	p-value (one-sided)
0.03197	0.11677	0.01569	4.30920	$9.12 \cdot 10^{-5}$

Despite a small sample size of $n_1 = 24$ and $n_2 = 8$, we find strong evidence for a trend change in the sense of a strongly significant increase of the slope parameter. Applying the test to the 28 European countries individually, we find the increase of the slope parameter to be significant in 21 of them. However, there is even stronger evidence.

1.3 Testing the equality of regression slopes – including the seasonal pattern

In very loose terms, t tests (and similar methods) compare the size of the quantity of interest (here the difference of two regression slopes) to the general variability present in the sample. Specifically, the above t test statistic contains the standard deviation of the residuals in the denominator. The remaining variability of the residuals is mainly the seasonal pattern (cp. Figure S1), which is systematic, and hence should not modelled as noise but rather incorporated in the model.

We fit a refined regression model with

$$Y^{(1)} = \begin{pmatrix} y_1 \\ y_2 \\ y_3 \\ y_4 \\ y_5 \\ y_6 \\ \vdots \\ y_{24} \end{pmatrix} = \begin{pmatrix} 1 & 1 & 0 & 0 & 0 \\ 1 & 2 & 1 & 0 & 0 \\ 1 & 3 & 0 & 1 & 0 \\ 1 & 4 & 0 & 0 & 1 \\ 1 & 5 & 0 & 0 & 0 \\ 1 & 6 & 1 & 0 & 0 \\ \vdots & \vdots & & & \\ 1 & 24 & 0 & 0 & 1 \end{pmatrix} \begin{pmatrix} \beta_{ic}^{(1)} \\ \beta_{sl}^{(1)} \\ \beta_{q2}^{(1)} \\ \beta_{q3}^{(1)} \\ \beta_{q4}^{(1)} \end{pmatrix} + \begin{pmatrix} \epsilon_1 \\ \epsilon_2 \\ \epsilon_3 \\ \epsilon_4 \\ \epsilon_5 \\ \epsilon_6 \\ \vdots \\ \epsilon_{24} \end{pmatrix} \tag{3}$$

for the pre-pandemic part and likewise for the post-pandemic part, assembled together analoguous to (2). Testing H_0: $\beta_{sl}^{(2)} \leq \beta_{sl}^{(1)}$ within this model, we obtain

$\hat{\beta}_{sl}^{(1)}$	$\hat{\beta}_{sl}^{(2)}$	$\hat{\sigma}^2$	t-value (test statistic)	p-value (one-sided)
0.03235	0.11294	0.00187	10.39083	$2.99 \cdot 10^{-10}$

We find the estimated slope coefficients have changed little when incorporating the seasonal pattern, but the residual variance is much smaller (see also Figure S1), hence the test statistic larger, and the p-value yet even smaller than before. Fitting this refined model to the 28 European countries individually and performing the test, we find the increase of the slope parameter to be significant in 26 of them, as reported in the paper.

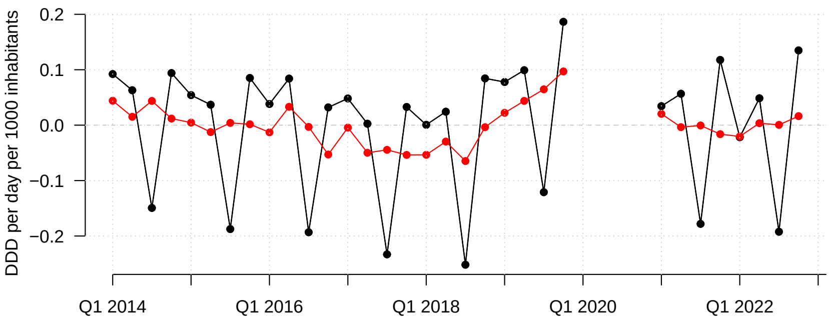

Figure S1: The residuals of the 'DDD per day and 1000 inhabitants' per quarter in Europe; based on the simple regression model (2) (black) and the refined model (3) incorporating the seasonal pattern (red).

Alternatively to fitting the seasonal patterns individually before and after the pandemic, one could have fit one seasonal pattern, i.e. having $(\beta_{q2}, \beta_{q3}, \beta_{q4})$ instead of $(\beta_{q2}^{(1)}, \beta_{q3}^{(1)}, \beta_{q4}^{(1)})$ and $(\beta_{q2}^{(2)}, \beta_{q3}^{(2)}, \beta_{q4}^{(2)})$. One can reason for both option, there is no strong reason against either, and the results are very similar.

2 Further Figures

2.1 Consumption levels in 28 European countries in 2019

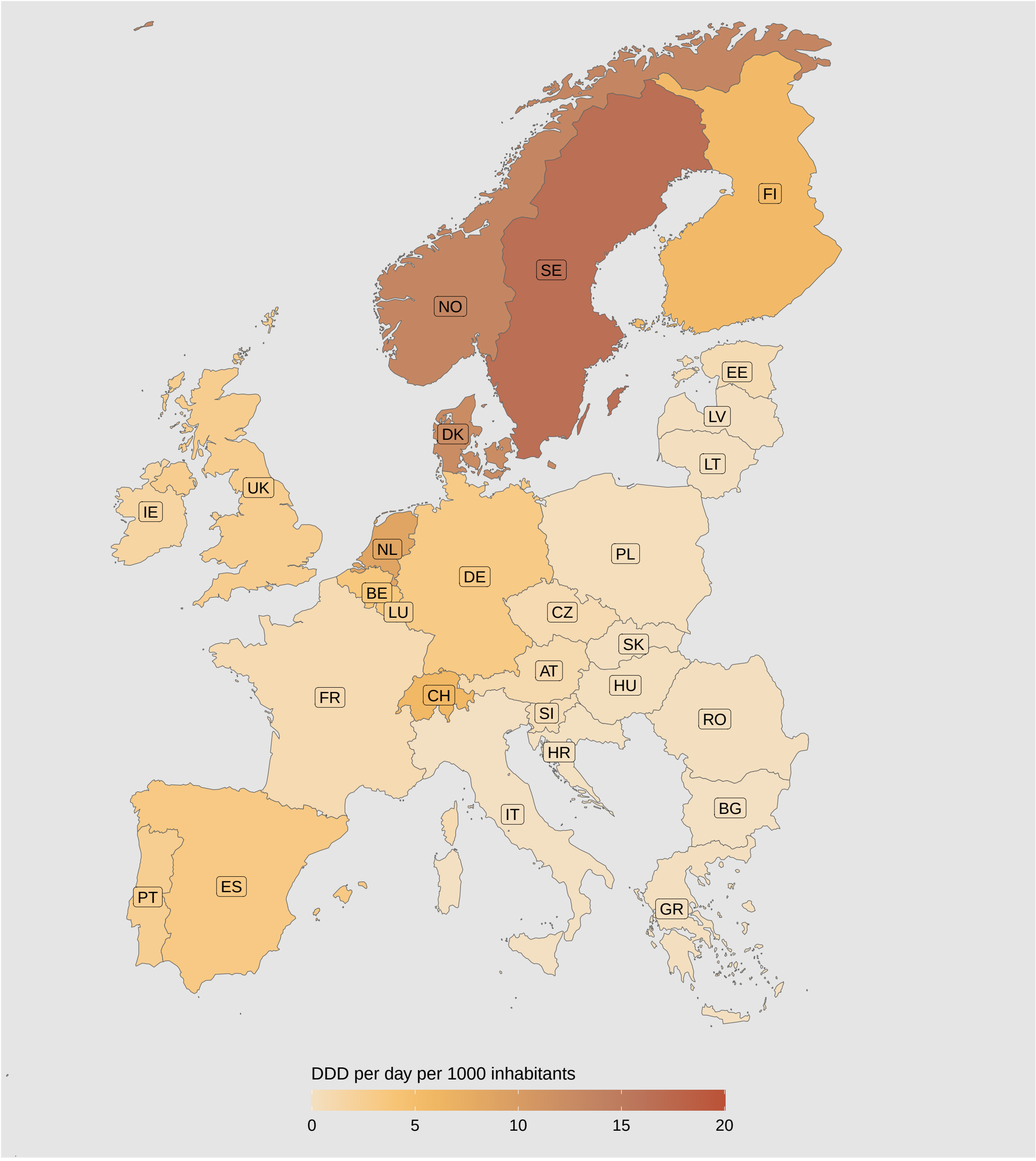

Figure S2: Consumption of ADHD medicines in 28 European countries, 2019. DDD = defined daily dose. Source: IQVIA MIDAS Quarterly Sales data Q1 2014 – Q4 2022.

2.2 Consumption growth in 28 European countries from 2014 to 2022

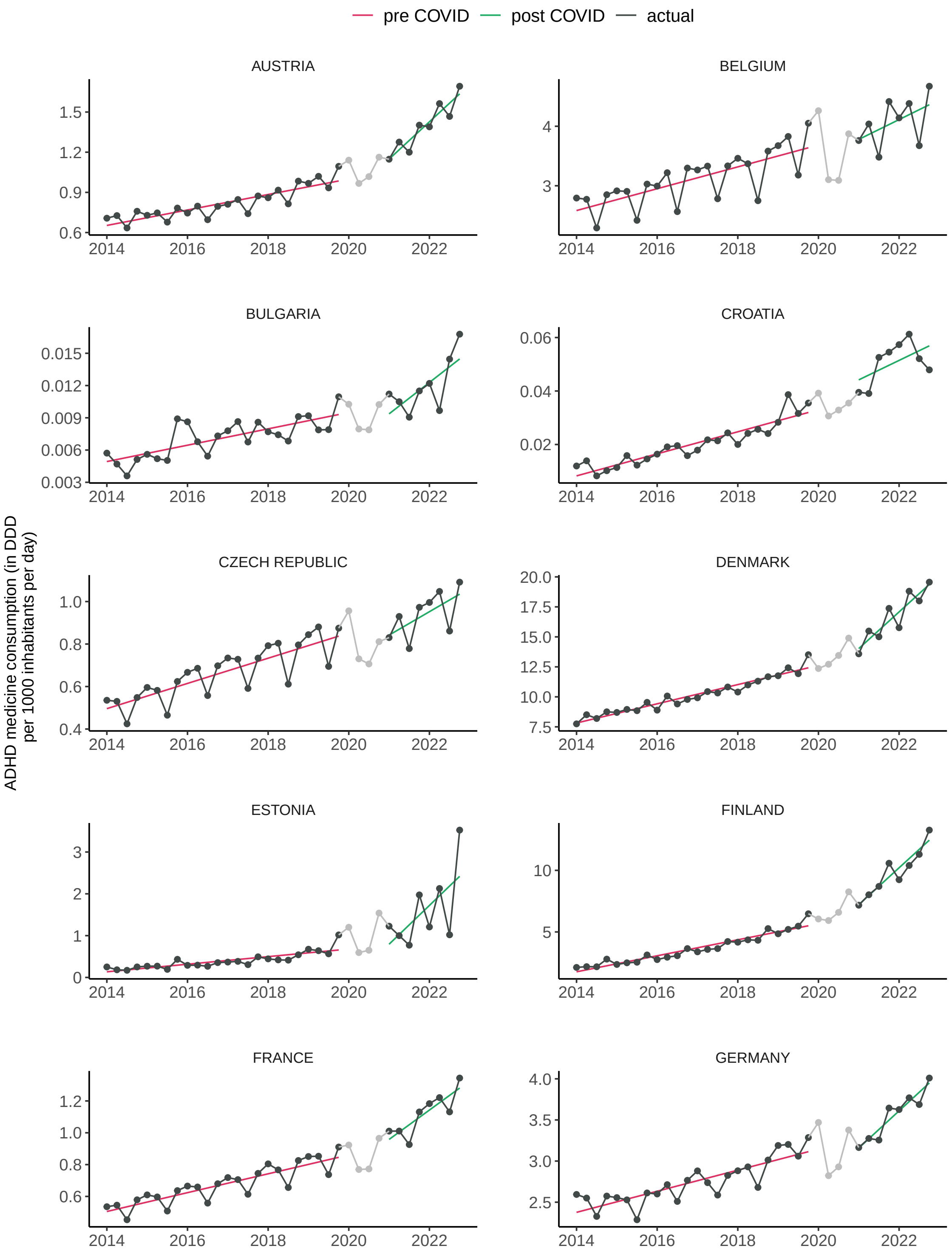

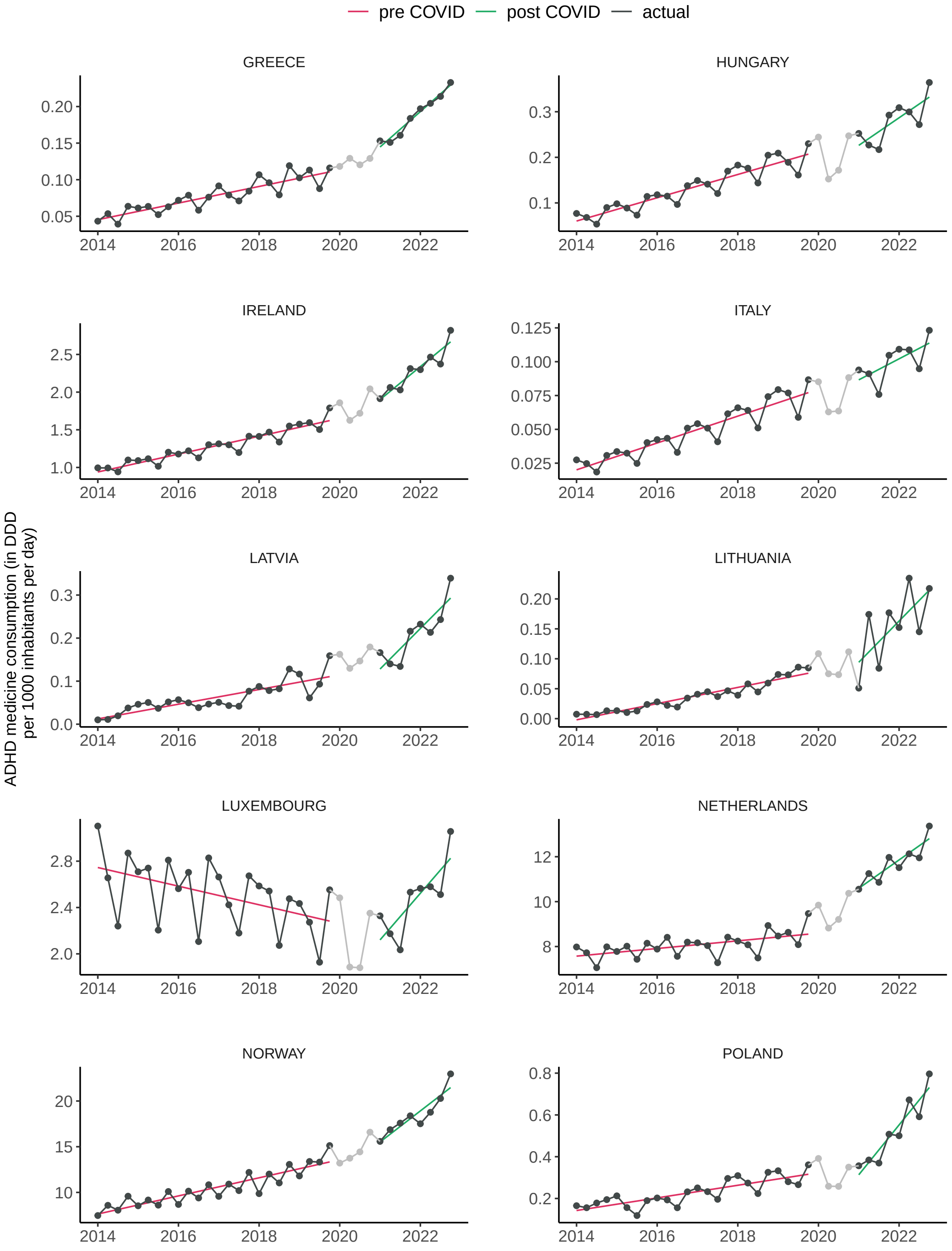

pre COVID
post COVID
actual
ADHD medicine consumption (in DDD per 1000 inhabitants per day)
GREECE
HUNGARY
IRELAND
ITALY
LATVIA
LITHUANIA
LUXEMBOURG
NETHERLANDS
NORWAY
POLAND
2014
2016
2018
2020
2022

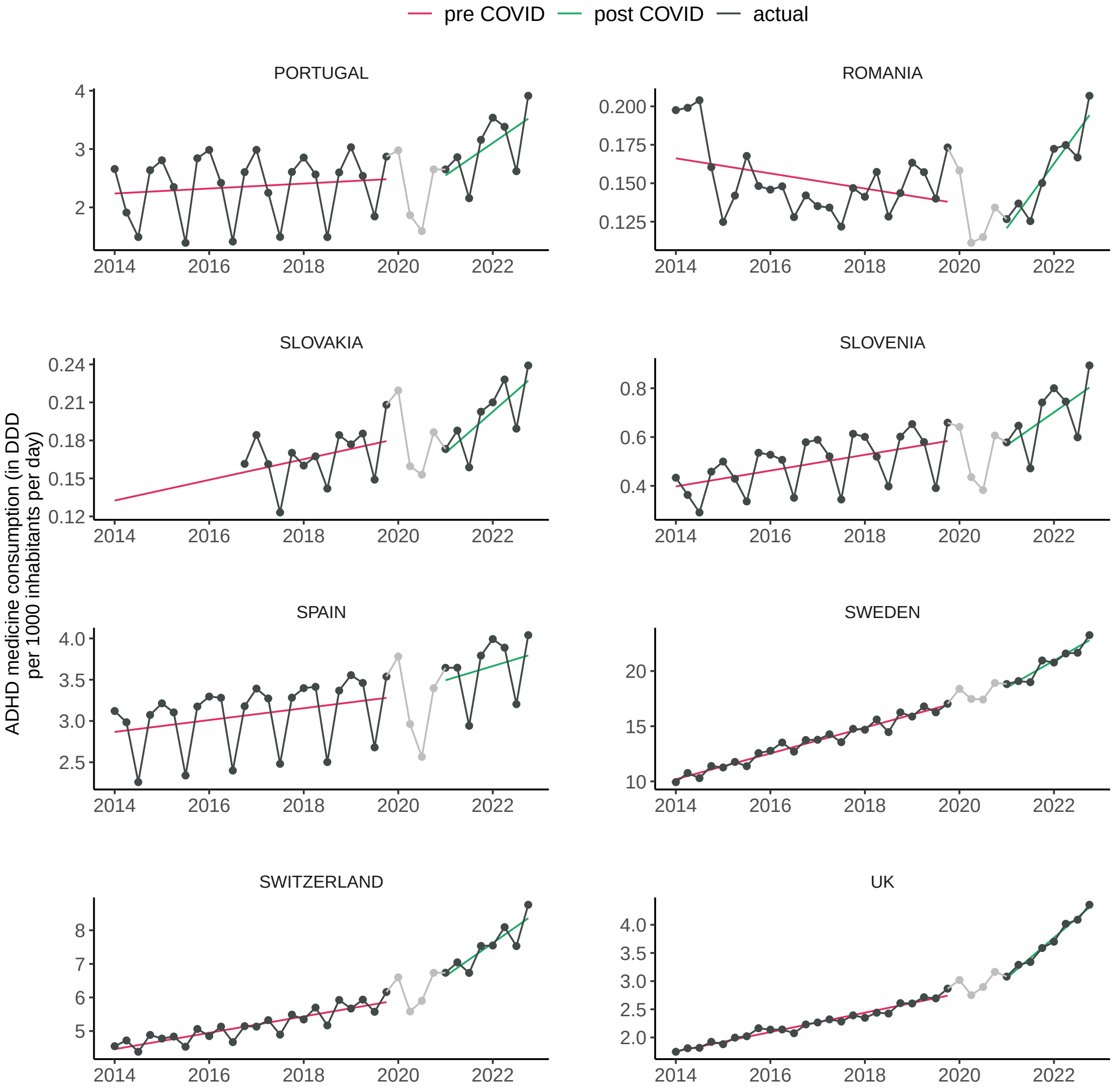

Figure S3: Evolution of ADHD medicine consumption in 28 European countries per quarter from 2014 to 2022. Linear trends were fitted by linear regression controlling for seasonal pattern pre-pandemic (2014–2019) and post-pandemic (2021–2022). DDD = defined daily dose. Source: IQVIA MIDAS Quarterly Sales data Q1 2014 – Q4 2022.

3 Data source

We used quarterly data obtained from IQVIA Multinational Integrated Data Analysis System (IQVIA MIDAS). IQVIA MIDAS data combine country-level data, healthcare expertise and therapeutic knowledge in 90+ countries to deliver data in globally standardized forms to facilitate multi-country analyses, a leading source of insight into international market dynamics relating to the distribution and use of medicines. IQVIA MIDAS data is designed to support multi-country analyses of trends, patterns and similar types of analyses. IQVIA national audits and MIDAS reflect local industry standard source of pack prices, which might be list price or average invoice price, depending upon the country and the available information; they do not reflect net prices realised by the manufacturers. Sales values reflected in these IQVIA audits are calculated by applying such relevant pricing to the product volume data collected for, and reflected in, such audits. In addition, to allow the national audit sales values to be viewed at a common sales level, MIDAS applies a single average industry margin to the locally reported values.

References

E. Lehmann and J. P. Romano. *Testing statistical hypotheses*. Springer Texts in Statistics. Springer, 3rd edition, 2005.
